# Vitamin D Receptor Gene Polymorphisms Modify Cardiometabolic Response to Vitamin D Supplementation in T2DM Patients

**DOI:** 10.1038/s41598-017-08621-7

**Published:** 2017-08-15

**Authors:** Nasser M. Al-Daghri, Abdul Khader Mohammed, Omar S. Al-Attas, Mohammed Ghouse Ahmed Ansari, Kaiser Wani, Syed D. Hussain, Shaun Sabico, Gyanendra Tripathi, Majed S. Alokail

**Affiliations:** 1 Prince Mutaib Chair for Biomarkers of Osteoporosis, Biochemistry Department, King Saud University, Riyadh, 11451 Saudi Arabia; 2 Biomarkers Research Program, Biochemistry Department, College of Science, King Saud University, Riyadh, 11451 Saudi Arabia; 3 Department of Biomedical Sciences, University of Westminster, London, W1W 6UW UK

## Abstract

There is conflicting evidence on the favorable effects of vitamin D supplementation on metabolic profile in Type 2 diabetes mellitus (T2DM) patients and this might be due to genetic variations in vitamin D receptors (VDRs). Thus, we studied the metabolic effects of a 12-month vitamin D supplementation in T2DM patients according to VDR polymorphisms. A total of 204 T2DM subjects received 2000 IU vitamin D3 daily for 12 months. Serum 25(OH)D and metabolic profiles were measured at baseline and after 12 months. VDR polymorphisms (*Taq-I, Bsm-I, Apa-I and Fok-I*) were identified using TaqMan genotyping assays. Vitamin D supplementation significantly increased HOMA β-cell function (p = 0.003) as well as significantly decreased triglycerides, total and LDL-cholesterol (p < 0.001). The lowest increment in 25(OH)D levels was detected in patients with *Fok-I* CC genotypes (p < 0.0001). With vitamin D supplementation, *Taq-I* GG genotype carriers showed significant improvements in triglycerides, LDL- and total cholesterol, insulin, HbA1c and HOMA-IR (p < 0.005, 0.01, < 0.001, < 0.005, 0.03 and 0.01, respectively). Similarly, *Bsm-I* TT genotype carriers showed significant improvements in triglycerides (p = 0.01), insulin and HOMA-IR (p-values < 0.05). In conclusion, improvements in metabolic profile due to vitamin D supplementation is influenced by VDR polymorphisms, specifically for carriers of *Taq-I* GG and *Bsm-I* TT genotypes.

## Introduction

Vitamin D plays a crucial role in calcium homeostasis and bone metabolism. Over the past decade, extra-skeletal effects of vitamin D has attracted considerable interest, including type 2 diabetes mellitus (T2DM)^1, 2^. *In vitro* studies showed that the active form of vitamin D (1,25-dihydroxyvitamin D or 1,25(OH)2D3) stimulates the release of insulin by the pancreatic β-cells^3, 4^. Numerous observational and epidemiological studies also reported an inverse association between serum 25-hydroxy vitamin D (25(OH)D) levels and fasting glucose, insulin resistance and lipid profile excluding HDL-cholesterol^5–10^. Despite promising results in favor of vitamin D, interventional studies yielded divergent results. Recent systematic reviews of randomized control trials (RCTs) in T2DM patients suggest an insufficient evidence about the beneficial effects of vitamin D supplementation on glucose homeostasis or insulin resistance^11, 12^. Similarly, some RCTs failed to indicate an association between vitamin D supplementation and a favorable lipid profile^13, 14^. In contrast, other studies have reported that vitamin D supplementation improved lipid profile and insulin sensitivity^15–18^. Differences in responses to vitamin D supplementation may partially be explained by the genetic makeup and variations involved in vitamin D pathway^19^. Vitamin D receptor (VDR) gene is one such factor responsible for regulating responses to vitamin D. It is known that the active form of vitamin D (1,25 (OH)2D3) binds with the cytosolic/nuclear VDR which then heterodimerizes with retinoid X receptor and regulates expression of several target genes^20^. In fact, VDR is expressed in a variety of tissues including muscles and pancreatic cells^21, 22^.

Several single nucleotide polymorphisms (SNPs) have been identified in the VDR gene including *Taq-I, Bsm-I, and Apa-I* polymorphisms, which are located at the 3′ untranslated region (3′ UTR) of the gene and are suspected to alter VDR expression^23, 24^. While another polymorphism known with *Fok-I* restriction site is located within the 5′ end near the promoter region^23^. *Fok-I* is a functional polymorphism that results in different translation initiation sites on VDR that lead to three amino acids becoming longer and less effective protein^25^. Since these polymorphisms affect the stability and activity of VDR mRNA and protein, it is likely that the individuals who are not responding cardiometabolically to vitamin D treatment might be due to these VDR SNPs. Indeed, recent studies have reported that the VDR genotypes may potentially affect the individual’s response to treatment. For instance, women with the VDR *Bsm-I* CC genotype had greater improvement in bone mineral density after treatment than those with the TT genotype^26^. Likewise, in patients with benign prostate hyperplasia, response to standard drug therapy is significantly associated with the VDR *Taq-I* genotype^27^.

There is limited information regarding the cardiometabolic effects of vitamin D intake based on VDR gene polymorphisms in T2DM subjects. The only study by Neyestani *et al*.^19^ have shown that subjects with VDR *Fok-I* ff genotype responded the least to vitamin D-fortified yogurt intake in terms of improvement serum 25(OH)D levels and some inflammatory markers. Given the potential of other VDR SNPs (*Taq-I, Bsm-I, Apa-I*) to influence the stability of VDR mRNA herein, we aim to evaluate the effects of vitamin D supplementation according to four different VDR SNPs (*Taq-I, Bsm-I, Apa-I and Fok-I*) on glycemic and lipid responses in T2DM patients. For this study, 204 T2DM patients who were mostly vitamin D deficient were enrolled and given 2000 IU vitamin D daily for 12 months, and screened for four different VDR SNPs (*Taq-I*, *Bsm-I*, *Apa-I and Fok-I*).

## Results

### Improved glycemic and lipid profile after 12 months of vitamin D supplementation

Table 1 shows the differences between baseline and 12-months supplementation in the studied subjects. There was a significant increase in serum 25(OH)D levels from baseline to12 months after vitamin D supplementation (p < 0.001). Improved serum 25(OH)D levels had a parallel significant decrease in LDL- total cholesterol (p < 0.0001) as well as triglycerides (p < 0.001) (Table 1). There was also a significant increase in serum insulin (p < 0.005) and HOMA-β cell function (p < 0.003).

**Table 1:** Anthropometrics and biochemical characteristics of all T2DM subjects at baseline and after 12 months of vitamin D supplementation.

N = 204	Baseline	Follow-up	*p* value
BMI (kg/m^2^)	32.3 ± 5.9	32.5 ± 6.0	0.008
Waist Circumference (cm)	106.2 ± 12.9	106.2 ± 12.9	0.843
Hips Circumference (cm)	110.9 ± 12.9	111.6 ± 13.7	0.112
Waist-Hip Ratio	1.0 ± 0.1	1.0 ± 0.1	0.581
Systolic Blood Pressure (mmHg)	127.0 ± 15.1	127.2 ± 14.5	0.897
Diastolic Blood Pressure (mmHg)	80.1 ± 9.2	79.2 ± 9.5	0.355
Triglycerides (mmol/l)	2.0 ± 0.8	1.9 ± 0.8	0.037
Total Cholesterol (mmol/l)	5.3 ± 1.1	4.9 ± 1.1	0.0001
HDL-Cholesterol (mmol/l)	1.0 ± 0.3	0.9 ± 0.3	0.0001
LDL-Cholesterol	3.3 ± 0.9	3.0 ± 1.0	0.0001
Fasting Glucose (mmol/l)	10.6 ± 4.6	10.7 ± 4.5	0.974
Insulin (uU/ml) #	15.8 ± 9.8	18.5 ± 12.0	0.005
HbA1c	7.5 ± 2.3	7.7 ± 2.5	0.274
HOMA-IR #	8.1 ± 7.9	8.7 ± 7.8	0.187
HOMA β-cell function #	60.3 ± 61.2	72.0 ± 67.4	0.003
25(OH)D (nmol/l)	33.4 ± 12.2	54.2 ± 17.6	0.0001

Note: Data were presented as mean ± standard deviation. # log-transformed prior to analysis. BMI- Body mass index; HOMA-IR- Homeostasis model assessment for insulin resistance.

### Effect of VDR genotypes on anthropometric and clinical parameters after vitamin D supplementation

**Figure 1:**
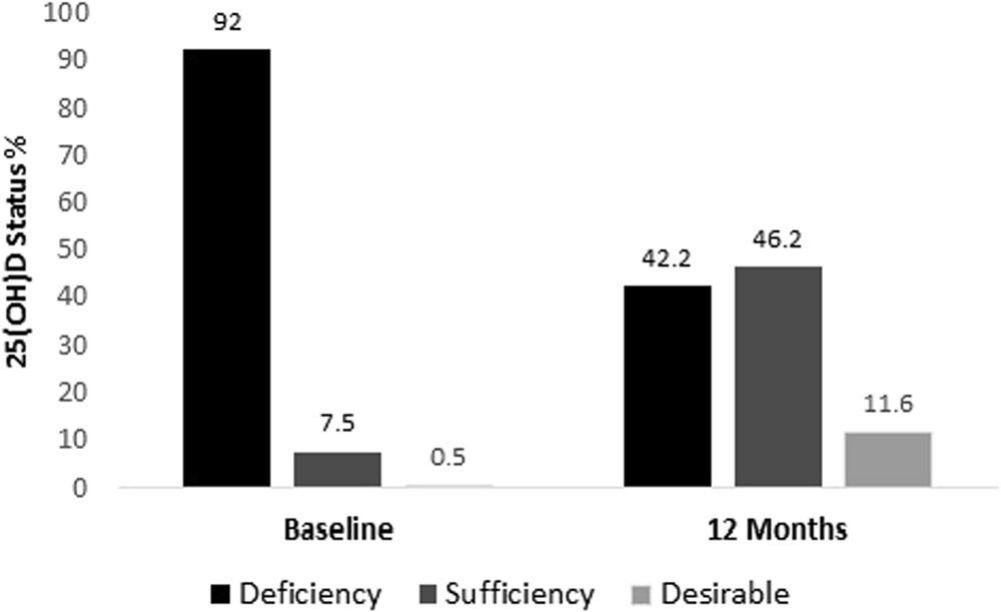
Comparison of the vitamin D status before and after 12 months of vitamin D supplementation. Deficiency: Serum 25(OH)D level < 50 nmol/L. Sufficiency: Serum 25(OH)D level ≥ 50 nmol/L. Desirable: Serum 25(OH)D level > 75 nmol/L.

**Figure 2:**
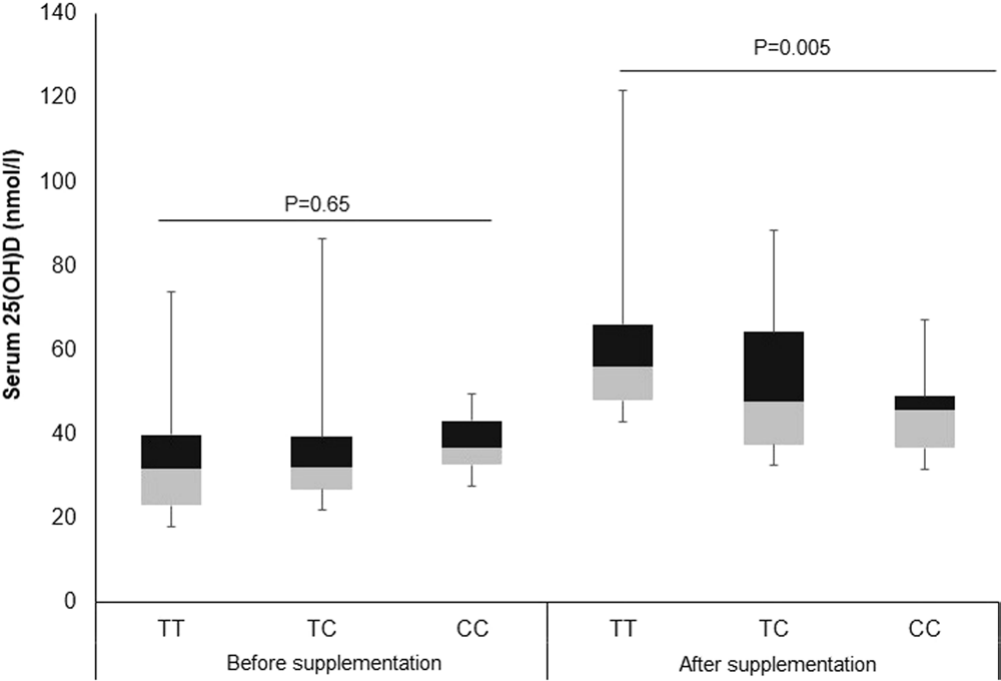
Box plot showing distribution of serum 25(OH)D levels (nmol/L) before and after vitamin D supplementation according to VDR *fok-I* genotypes.

Prevalence of vitamin D sufficiency (serum 25(OH)D > 50 nmol/L) increased after 12 months of vitamin D supplementation. However, around 42.2% of the subjects remained vitamin D deficient (Fig. 1). To explore whether VDR genotype has any influence on determining the vitamin D status (Deficiency/Sufficiency), subjects were compared according to VDR genotype. At baseline, there was no significant trend in distribution of vitamin D deficiency status among VDR *Fok-I* variants (P-trend = 0.626). However, after intervention, trend test was highly significant (P-trend < 0·0001), showing > 51% of subjects with *Fok-I* TC genotype and almost all subjects with CC genotype remained vitamin D deficient (Table 2). Further data analysis indicates that after 12 months of vitamin D supplementation, subjects in *Fok-I* TT group showed better improvement (increased) in serum 25(OH)D levels compared to CC group (TT 24.4 ± 15.8 *vs* CC 8.4 ± 9.6; p < 0.001) (Table 3 and Fig. 2). This association remained significant (p = 0.005) even after adjusting for confounders such as age, gender, BMI and baseline 25(OH)D levels. After Bonferroni correction, this association remained significant (P = 0.02). In addition, subjects with the rare *Fok-I* CC genotype also showed significant increase in BMI (1.0 ± 1.4 kg/m2, p = 0.02) compared to TT and TC genotypes, after 12 months of vitamin D supplementation (Table 3).

**Table 2:** Vitamin D status among the *Fok-I* genotypic groups before and after 12 months of vitamin D supplementation.

Vitamin D status (N = 199)	TT (N = 113)		TC (70)		CC (16)	
	Baseline	Follow-up	Baseline	Follow-up	Baseline	Follow-up
Desirable	0 (0.0)	14 (12.4)	1 (1.4)	9 (12.9)	0 (0.0)	0 (0.0)
Sufficiency	10 (8.8)	66 (58.4)	4 (5.7)	25 (35.7)	1 (6.3)	1 (6.3)
Deficiency	103 (91.2)	33 (29.2)	65 (92.9)	36 (51.4)	15 (93.8)	15 (93.8)

Note: Σ^2^ test showed a P_trend_ of 0.626 at baseline; P_trend_ at 12-month follow-up is < 0.0001. Data is presented as frequencies (%). Deficiency: Serum 25(OH)D level < 50 nmol/l. Sufficiency: Serum 25(OH)D level between 50 to 75 nmol/l. Desirable: Serum 25(OH)D level > 75 nmol/l.

**Table 3:** Comparisons of the variables among the *Fok-I* genotypic before and after 12 months of vitamin D supplementation.

Variables		TT (113)	TC (70)	CC (16)	P value	P^a^
BMI (kg/m^2^)	Baseline	32.1 ± 5.9	32.4 ± 6.2	32.5 ± 5.4	**0.02**	0.01*
	12 Months	32.4 ± 6.0	32.5 ± 6.2	33.6 ± 5.2		
	Change	0.2 ± 0.9	0.1 ± 1.1	1.0 ± 1.4^aa^		
Systolic BP (mmHg)	Baseline	126.7 ± 16.0	128.7 ± 13.9	116.0 ± 8.9	0.10	0.07
	12 Months	127.5 ± 14.6	126.4 ± 14.8	128.0 ± 11.0		
	Change	0.9 ± 14.9	−2.2 ± 14.1	12.0 ± 11.0		
Diastolic BP (mmHg)	Baseline	79.7 ± 8.9	81.8 ± 9.1	70.0 ± 7.1	0.16	0.26
	12 Months	79.5 ± 8.9	79.1 ± 10.1	76.0 ± 13.4		
	Change	−0.3 ± 11.0	−2.7 ± 9.2	6.0 ± 18.2		
Triglycerides (mmol/l)	Baseline	2.0 ± 0.8	1.9 ± 0.8	2.0 ± 1.2	0.49	0.49
	12 Months	1.8 ± 0.8	1.9 ± 0.9	1.8 ± 0.9		
	Change	−0.1 ± 0.7	0.0 ± 0.7	−0.2 ± 0.7		
Total Cholesterol (mmol/l) (mmol/l)	Baseline	5.3 ± 1.0	5.4 ± 1.1	5.0 ± 1.1	0.94	0.63
	12 Months	4.9 ± 1.0	5.0 ± 1.2	4.7 ± 1.0		
	Change	−0.4 ± 0.9	−0.4 ± 1.0	−0.3 ± 1.2		
HDL-Cholesterol (mmol/l)	Baseline	1.0 ± 0.3	1.1 ± 0.3	0.9 ± 0.4	0.47	0.14
	12 Months	0.9 ± 0.3	0.9 ± 0.3	0.9 ± 0.4		
	Change	−0.1 ± 0.3	−0.2 ± 0.3	0.0 ± 0.5		
LDL-Cholesterol (nmol/l)	Baseline	3.3 ± 0.8	3.4 ± 1.1	3.0 ± 0.7	0.38	0.59
	12 Months	3.0 ± 0.9	3.0 ± 1.2	2.9 ± 0.9		
	Change	−0.3 ± 0.9	−0.4 ± 1.0	−0.1 ± 0.8		
Glucose (mmol/l)	Baseline	10.1 ± 3.9	11.9 ± 5.1	8.7 ± 5.0	0.33	0.12
	12 Months	10.5 ± 4.1	11.4 ± 5.1	8.9 ± 3.6		
	Change	0.4 ± 3.9	−0.6 ± 4.9	0.2 ± 4.4		
Insulin (uU/ml)	Baseline	16.1 ± 9.6	15.7 ± 10.2	12.6 ± 9.8	0.15	0.19
	12 Months	17.9 ± 11.7	18.9 ± 13.0	22.3 ± 5.6		
	Change	1.8 ± 12.1	3.2 ± 10.6	9.7 ± 11.3		
HbA1c	Baseline	7.1 ± 2.1	8.1 ± 2.5	6.0 ± 1.7	0.28	0.25
	12 Months	7.5 ± 2.4	8.0 ± 2.6	6.8 ± 1.9		
	Change	0.4 ± 2.2	−0.2 ± 2.7	0.8 ± 2.5		
HOMA-IR	Baseline	8.1 ± 8.7	8.3 ± 6.9	4.9 ± 3.0	0.14	0.14
	12 Months	8.2 ± 8.1	9.4 ± 7.4	10.0 ± 7.4		
	Change	0.1 ± 5.9	1.0 ± 6.6	5.2 ± 6.3		
25(OH)D (nmol/l)	Baseline	33.0 ± 12.4	33.6 ± 12.3	36.0 ± 10.0	**0.001**	0.005**
	12 Months	57.4 ± 17.3	51.3 ± 18.4	44.5 ± 9.2		
	Change	24.4 ± 15.8	17.7 ± 19.1	8.4 ± 9.6^a^		

Note: Change = Follow up − Baseline; p values presented are for the changes in different variables after vitamin D supplementation according to *Fok-I* genotypes; P^a^ indicates p values after adjusting age, gender, BMI and baseline 25(OH)D levels; Superscript “a” indicates significantly different from TT group after post hoc analysis; “*” indicates p < 0.05 after Bonferroni correction.

Next, we compared the changes in glycemic and lipid profile according to VDR genotypes after intervention. Subjects with *Taq-I* GG genotype group showed significantly better improvements in glucose and lipid profile than other genotypes. A significant decrease in total cholesterol (−0.9 ± 0.9 mmol/l, p < 0.001), LDL-cholesterol (−0.7 ± 1.1, p = 0.01), triglycerides (−0.4 ± 0.5, p < 0.005), insulin (−2.3 ± 12.0 uU/ml, p < 0.005), HbA1c (p = 0.03) and HOMA-IR (p = 0.01) were observed in subjects with GG genotype than subjects with AA and AG genotypes (Table 4). However, significance for LDL-cholesterol (p = 0.09) and HOMA-IR (0.08) disappeared after adjustments for age, gender, BMI and baseline vitamin D levels. Associations of *Taq-I* genotypes with triglycerides, total-cholesterol, insulin and HbA1C remained significant after Bonferroni correction. Similarly, subjects carrying *Bsm-I* CC genotypes had better improvements in triglycerides (−0.4 ± 0.5, p = 0.01), insulin (−2.0 ± 11.7 uU/ml, p = 0.01) and HOMA-IR (p = 0.05) than other genotypes (Table 5). However, significance for HOMA-IR disappeared after adjustment with confounding factors. Likewise, subjects carrying *Apa-I* heterozygous AC genotype showed significantly better improvement in systolic blood pressure (−4.3 ± 14.5 mmHg, p = 0.009) than with AA and CC genotypes (Table 6).

**Table 4:** Comparisons of the variables among the *Taq-I* genotypic before and after 12 months of vitamin D supplementation.

Variables		GG (46)	AG (99)	AA (54)	P Value	P^a^
BMI (kg/m^2^)	Baseline	32.0 ± 6.3	31.7 ± 5.9	33.5 ± 5.7	0.21	0.18
	12 Months	32.1 ± 6.2	31.9 ± 6.0	33.9 ± 5.7		
	Change	0.1 ± 0.9	0.1 ± 1.1	0.4 ± 0.9		
Systolic BP (mmHg)	Baseline	128.6 ± 17.3	126.3 ± 14.3	126.9 ± 15.1	0.58	0.35
	12 Months	130.9 ± 16.8	126.3 ± 13.0	125.2 ± 14.8		
	Change	2.3 ± 15.4	0.1 ± 15.8	−1.7 ± 11.0		
Diastolic BP (mmHg)	Baseline	80.7 ± 10.0	80.0 ± 8.4	79.7 ± 10.2	0.21	0.20
	12 Months	82.0 ± 9.8	79.3 ± 9.4	76.0 ± 8.8		
	Change	1.3 ± 11.3	−0.7 ± 11.4	−3.6 ± 8.3		
Triglycerides (mmol/l)	Baseline	2.0 ± 0.9	2.0 ± 0.9	1.9 ± 0.8	**0.005**	0.01*
	12 Months	1.6 ± 0.9	1.9 ± 0.8	1.9 ± 0.8		
	Change	−0.4 ± 0.5	−0.1 ± 0.7^a^	0.0 ± 0.7		
Total Cholesterol (mmol/l)	Baseline	5.3 ± 1.1	5.5 ± 1.0	5.0 ± 1.1	**0.001**	0.01*
	12 Months	4.4 ± 0.9	5.2 ± 1.0	4.9 ± 1.1		
	Change	−0.9 ± 0.9	−0.3 ± 0.9	−0.1 ± 0.9^a^		
HDL-Cholesterol (mmol/l)	Baseline	1.0 ± 0.3	1.0 ± 0.3	1.0 ± 0.3	0.22	0.71
	12 Months	0.8 ± 0.3	0.9 ± 0.3	1.0 ± 0.3		
	Change	−0.1 ± 0.4	−0.2 ± 0.3	−0.1 ± 0.3		
LDL-Cholesterol (nmol/l)	Baseline	3.4 ± 1.0	3.4 ± 0.9	3.1 ± 1.0	**0.01**	**0.09**
	12 Months	2.7 ± 0.8	3.2 ± 1.1	2.9 ± 0.9		
	Change	−0.7 ± 1.1	−0.2 ± 0.9	−0.2 ± 0.8		
Glucose (mmol/l)	Baseline	11.0 ± 3.8	10.6 ± 4.6	10.5 ± 5.1	0.06	0.14
	12 Months	9.8 ± 4.1	10.8 ± 4.5	11.2 ± 4.6		
	Change	−1.2 ± 3.3	0.2 ± 4.5	0.8 ± 4.6		
Insulin (uU/ml)	Baseline	19.0 ± 10.2	15.7 ± 9.9	13.4 ± 8.8	**0.005**	0.01*
	12 Months	16.7 ± 10.3	19.1 ± 11.8	18.8 ± 13.9		
	Change	−2.3 ± 12.0	3.4 ± 11.4^a^	5.4 ± 10.3^a^		
HbA1c	Baseline	7.9 ± 2.4	7.2 ± 2.1	7.6 ± 2.5	**0.03**	0.01*
	12 Months	7.2 ± 2.8	7.7 ± 2.3	8.0 ± 2.5		
	Change	−0.7 ± 2.2	0.5 ± 2.4^a^	0.4 ± 2.5^a^		
HOMA-IR	Baseline	9.2 ± 5.5	8.4 ± 9.8	6.6 ± 5.0	**0.01**	**0.04**
	12 Months	7.6 ± 6.5	9.5 ± 8.9	8.4 ± 6.4		
	Change	−1.6 ± 5.5	1.1 ± 6.9	1.9 ± 5.1		
25(OH)D (nmol/l)	Baseline	31.9 ± 12.7	34.4 ± 12.6	33.3 ± 10.7	0.56	0.62
	12 Months	51.2 ± 13.6	54.7 ± 20.2	56.1 ± 15.5		
	Change	19.2 ± 15.7	20.3 ± 18.9	22.8 ± 15.4		

Note: Change = Follow up − Baseline; p values presented are for the changes in different variables after vitamin D supplementation according to *Taq-I* genotypes; P^a^ indicates p values after adjusting age, gender, BMI and baseline 25(OH)D levels; Superscript “a” indicates significantly different from GG group after post hoc analysis; “*” indicates p < 0.05 after Bonferroni correction.

**Table 5:** Comparisons of the variables among the *Bsm-I* genotypic before and after 12 months of vitamin D supplementation.

Variables		TT (41)	CT (103)	CC (55)	P value	P^a^
BMI (kg/m^2^)	Baseline	32.2 ± 6.1	31.4 ± 5.5	34.1 ± 6.3	0.23	0.17
	12 Months	32.4 ± 6.0	31.5 ± 5.6	34.6 ± 6.2		
	Change	0.2 ± 0.9	0.1 ± 1.1	0.4 ± 0.9		
Systolic BP (mmHg)	Baseline	128.6 ± 17.3	125.5 ± 14.0	128.6 ± 15.6	0.22	0.09
	12 Months	130.9 ± 16.8	126.6 ± 12.8	124.6 ± 15.3		
	Change	2.3 ± 15.4	1.0 ± 15.0	−3.9 ± 12.9		
Diastolic BP (mmHg)	Baseline	80.7 ± 10.0	79.5 ± 8.4	80.7 ± 10.2	0.07	0.10
	12 Months	82.0 ± 9.8	79.3 ± 9.3	75.9 ± 8.9		
	Change	1.3 ± 11.3	−0.2 ± 11.1	−4.8 ± 8.6		
Triglycerides (mmol/l)	Baseline	3.2 ± 1.0	3.4 ± 0.9	3.2 ± 1.0	0.01	0.03*
	12 Months	2.8 ± 0.9	3.2 ± 1.1	2.8 ± 0.9		
	Change	−0.5 ± 1.2	−0.2 ± 0.9^a^	−0.4 ± 0.9		
Total Cholesterol (mmol/l)	Baseline	5.2 ± 1.1	5.5 ± 1.0	5.1 ± 1.0	0.27	0.17
	12 Months	4.6 ± 1.0	5.2 ± 1.0	4.7 ± 1.0		
	Change	−0.6 ± 1.1	−0.3 ± 0.9	−0.4 ± 0.9		
HDL-Cholesterol (mmol/l)	Baseline	1.0 ± 0.3	1.0 ± 0.3	1.0 ± 0.3	0.32	0.44
	12 Months	0.9 ± 0.3	0.9 ± 0.3	0.9 ± 0.3		
	Change	−0.1 ± 0.3	−0.2 ± 0.3	−0.1 ± 0.4		
LDL-Cholesterol (nmol/l)	Baseline	2.1 ± 0.8	2.0 ± 0.8	1.9 ± 0.8	0.38	0.39
	12 Months	1.7 ± 0.9	1.9 ± 0.8	1.8 ± 0.8		
	Change	−0.4 ± 0.5	0.0 ± 0.7	−0.1 ± 0.7		
Glucose (mmol/l)	Baseline	11.1 ± 3.7	10.5 ± 4.6	10.6 ± 5.0	0.30	0.41
	12 Months	10.2 ± 4.2	10.9 ± 4.6	10.7 ± 4.5		
	Change	−0.9 ± 3.7	0.3 ± 4.4	0.1 ± 4.6		
Insulin (uU/ml)	Baseline	18.6 ± 10.1	15.7 ± 9.9	13.6 ± 9.0	**0.01**	0.01*
	12 Months	16.6 ± 10.0	19.3 ± 11.7	18.7 ± 14.2		
	Change	−2.0 ± 11.7	3.6 ± 11.3^a^	5.1 ± 10.8^a^		
HbA1c	Baseline	8.0 ± 2.3	7.1 ± 2.2	7.5 ± 2.5	0.06	0.02*
	12 Months	7.5 ± 2.8	7.7 ± 2.4	7.7 ± 2.5		
	Change	−0.5 ± 2.3	0.6 ± 2.3^a^	0.2 ± 2.6		
HOMA-IR	Baseline	9.0 ± 5.4	8.4 ± 9.8	6.6 ± 5.1	0.05	0.10
	12 Months	7.7 ± 6.4	9.6 ± 8.9	8.0 ± 6.5		
	Change	−1.2 ± 5.5	1.2 ± 6.9	1.4 ± 5.2		
25(OH)D (nmol/l)	Baseline	31.1 ± 14.0	34.5 ± 12.4	33.6 ± 9.8	0.47	0.41
	12 Months	50.1 ± 14.7	54.6 ± 19.8	56.6 ± 14.9		
	Change	19.0 ± 15.9	20.2 ± 18.5	23.0 ± 15.9		

Note: Change = Follow up − Baseline; p values presented are for the changes in different variables after vitamin D supplementation according to *Bsm-I* genotypes; P^a^ indicates p values after adjusting age, gender, BMI and baseline 25(OH)D levels; Superscript “a” indicates significantly different from TT group after post hoc analysis; “*” indicates p < 0.05 after Bonferroni correction.

**Table 6:** Comparisons of the variables among the *Apa-I* genotypic before and after 12 months of vitamin D supplementation.

Variables		AA (87)	AC (84)	CC (28)	P Value	P^a^
BMI (kg/m^2^)	Baseline	33.7 ± 5.6	32.2 ± 6.2	31.9 ± 5.7	0.47	0.51
	12 Months	34.2 ± 5.4	32.4 ± 6.3	32.1 ± 5.8		
	Change	0.5 ± 1.0	0.1 ± 0.9	0.2 ± 1.1		
Systolic BP (mmHg)	Baseline	126.9 ± 11.8	130.1 ± 16.3	124.1 ± 14.4	**0.009**	0.001*
	12 Months	127.7 ± 10.9	125.8 ± 15.4	128.3 ± 14.4		
	Change	0.8 ± 10.4	−4.3 ± 14.5^a^	4.2 ± 14.7		
Diastolic BP (mmHg)	Baseline	80.8 ± 9.5	81.2 ± 8.9	78.9 ± 9.3	0.05	0.07
	12 Months	76.2 ± 9.6	78.4 ± 9.1	80.6 ± 9.8		
	Change	−4.6 ± 6.6	−2.7 ± 10.6	1.6 ± 11.3		
Triglycerides (mmol/l)	Baseline	1.9 ± 0.7	1.9 ± 0.8	2.0 ± 0.9	0.50	0.98
	12 Months	1.8 ± 0.8	1.9 ± 0.7	1.8 ± 0.9		
	Change	−0.1 ± 0.7	0.0 ± 0.7	−0.2 ± 0.7		
Total Cholesterol (mmol/l)	Baseline	5.1 ± 1.1	5.4 ± 0.9	5.3 ± 1.1	0.99	0.93
	12 Months	4.7 ± 1.0	5.0 ± 1.1	4.9 ± 1.0		
	Change	−0.4 ± 1.0	−0.4 ± 1.0	−0.4 ± 0.9		
HDL-Cholesterol (mmol/l)	Baseline	1.0 ± 0.3	1.0 ± 0.3	1.0 ± 0.3	0.90	0.98
	12 Months	0.9 ± 0.4	0.9 ± 0.3	0.8 ± 0.3		
	Change	−0.1 ± 0.5	−0.1 ± 0.3	−0.2 ± 0.3		
LDL-Cholesterol (nmol/l)	Baseline	3.0 ± 0.9	3.5 ± 0.8	3.2 ± 1.0	0.79	0.77
	12 Months	2.7 ± 0.9	3.1 ± 1.0	2.9 ± 1.0		
	Change	−0.3 ± 0.7	−0.4 ± 1.0	−0.3 ± 1.0		
Glucose (mmol/l)	Baseline	11.0 ± 5.9	10.7 ± 4.3	10.6 ± 4.3	0.69	0.62
	12 Months	10.4 ± 4.2	10.8 ± 4.6	10.6 ± 4.4		
	Change	−0.6 ± 4.9	0.2 ± 4.7	0.1 ± 3.7		
Insulin (uU/ml)	Baseline	14.4 ± 7.0	15.3 ± 10.1	16.8 ± 10.2	0.98	0.86
	12 Months	17.0 ± 11.6	18.3 ± 13.1	19.1 ± 11.1		
	Change	2.6 ± 7.9	3.0 ± 11.7	2.3 ± 12.4		
HbA1c	Baseline	7.4 ± 2.7	7.4 ± 2.3	7.5 ± 2.2	0.90	0.83
	12 Months	7.8 ± 2.5	7.6 ± 2.4	7.7 ± 2.6		
	Change	0.4 ± 3.0	0.2 ± 2.3	0.1 ± 2.4		
HOMA-IR	Baseline	6.7 ± 3.5	7.9 ± 6.6	8.7 ± 9.9	0.63	0.66
	12 Months	6.6 ± 3.4	8.5 ± 7.0	9.7 ± 9.3		
	Change	−0.2 ± 2.9	0.6 ± 6.6	1.0 ± 6.6		
25(OH)D (nmol/l)	Baseline	35.1 ± 9.5	35.0 ± 13.6	31.7 ± 11.2	0.97	0.44
	12 Months	56.2 ± 13.3	55.9 ± 20.3	52.1 ± 16.1		
	Change	21.1 ± 14.3	20.9 ± 18.2	20.4 ± 17.4		

Note: Change = Follow up − Baseline; p values presented are for the changes in different variables after vitamin D supplementation according to *Apa-I* genotypes; P^a^ indicates p values after adjusting age, gender, BMI and baseline 25(OH)D levels; Superscript “a” indicates significantly different from AA group after post hoc analysis; “*” indicates p < 0.05 after Bonferroni correction.

### Haplotype and linkage disequilibrium (LD) analysis

VDR gene polymorphisms at *Taq-I* were in LD with both *Bsm-I* and *Apa-I* (R2 = 0.87 and −0.44 respectively). In addition, *Bsm-I* and *ApaI* SNPs were also in LD with each other (R2 = −0.61) (Supplementary Table S1). As there was a strong positive LD between *Taq-I* and *Bsm-I* SNPs, indicating two alleles occurred on the same haplotype more than expected. Thus, we combined the different genotypes from *Taq-I* and *Bsm-I* to check the haplotype effect on various parameters. After 12 months of vitamin D supplementation, *Taq-I*GG + *Bsm-I*TT haplotype showed significantly higher decrease in total cholesterol (−0.8 ± 0.9; p = 0.015), glucose (−1.3 ± 3.6; p = 0.036), insulin (−2.3 ± 12.0; p = 0.010), triglycerides (−0.4 ± 0.5; p = 0.012), HbA1c (−0.7 ± 2.3; p = 0.03) and HOMA-IR (−1.6 ± 5.5; p = 0.024) compared to *Taq-I*AA + *Bsm-I*CC and *Taq-I*AG + *Bsm-I*CG (Supplementary Table S2).

## Disscussion

The present study showed varying degrees of metabolic improvements from vitamin D supplementation, partly due to variability in the VDR gene. In patients with *Fok-I* CC genotypic group, improvement in serum 25(OH)D levels was the least compared to other *Fok-I* genotypic groups, implying that VDR gene polymorphisms might be crucial for vitamin D intervention. Intake of 2000 IU/D vitamin D has beneficial effects on glycemic and lipid profile, but these effects were more pronounced in patients with *Taq-I* GG and *Bsm-I* TT genotypes in VDR gene.

In the present study, 42.2% of the subjects were not able to achieve the desirable 25(OH)D levels (>50 nmol/L) despite a year-long supplementation. In a RCT on obese T2DM Emirati participants, receiving high doses vitamin D3 was not sufficient to achieve target serum 25(OH)D levels (≥75 nmol/L)^28^. In a pilot study on American subjects randomized to 800 IU/d of supplemental vitamin D, only 50% of the participants reached the desirable serum 25(OH)D levels^29^. Moreover, our previous data in Saudi T2DM patients treated with 2000 IU/D of vitamin D for 18 months, as much as 22% of subjects remained deficient^30^. Since genetic analyses was missing in these studies, variations in the genes that regulate 25(OH)D levels could be one explanation. To prove this, data from the present study indicated an unaltered vitamin deficiency status in subjects belonging to VDR *Fok-I* CC group. Individuals with homozygous major allele *Fok-I* (TT) had significantly better increase in serum 25(OH)D levels than CC genotype. It is likely that causal variant (*Fok-I* CC) could possibly alter their role in metabolic feedback loops or effect the speed at which 25(OH)D is metabolized^31^. Smolders *et al*. have reported that 1,25(OH)2D limits its own levels and the levels of its precursor 25(OH)D via the VDR and demonstrated that 25(OH)D and 1,25(OH)2D-hydroxylation is affected by the *Fok-I* VDR polymorphism^32^. However, these genotypic variants were not associated with baseline 25(OH)D levels in the present study. It can be speculated that *Fok-I* genotypic variants might require a higher threshold 25(OH)D levels to exhibit its feedback effects. Alternatively, this SNP may be in linkage disequilibrium with other neighboring functional polymorphisms which are known to affect baseline 25(OH)D levels. Perhaps, there could be different mechanisms that regulate 25(OH)D derived from supplementation vs cutaneous synthesis. In agreement with our results, Neyestani *et al*. reported T2DM patients with minor *Fok-I* ff (corresponds to *Fok-I* CC in current study) had significantly lower serum 25(OH)D levels after daily intake of vitamin D fortified yoghurt^19^. Similarly, in a randomized double blinded, placebo-controlled trial, Yao *et al*. demonstrated an association between *Fok-I* G allele and increase in 25(OH)D levels^33^. Data form previous studies and non-genetic factors including age, BMI and baseline 25(OH)D concentrations could influence vitamin D treatment outcome^34, 35^. The association between *Fok-I* genotypes and increase in serum 25(OH)D levels remained significant even after adjustments of the above mentioned confounding factors, indicating an independent association.

Longitudinal studies have indicated an association between elevated serum 25(OH)D levels with lower T2DM risk^8, 36^. However, results from various RCTs have yielded conflicting results. The current study suggests that vitamin D intake favorably affects insulin sensitivity and lipid profile of T2DM patients. Muñoz-Aguirre *et al*.^15^ have also reported that in T2DM patients, 4000 IU/D of vitamin D intake may improve serum triglycerides. Similarly, Pittas *et al*. reported that in the elderly people with impaired fasting glucose, vitamin D and calcium supplementation might attenuate insulin resistance^37^. On the contrary, a recent systematic review and meta-analysis by Sieda *et al*. involving 35 RCTs concluded no effect of vitamin D supplementation on glucose homeostasis or diabetes prevention^11^. One possible reason for varying results could be the heterogeneity of the studied cohorts and the genetic differences among various ethnic groups. Thus, studying the genetic polymorphisms involved in vitamin D pathway might elucidate the potential reasons for inconsistent responses to vitamin D supplementation. For instance, the frequency of VDR *Taq-I* GG was 19.2% in the current study, while it was 15.6% in Non-Hispanic whites, 7.2% in Non-Hispanic blacks and 4.9% in Mexican-Americans^38^. Vitamin D modulates the expression of target genes upon binding to VDR, thus studying SNPs in VDR gene might provide insightful inter-individual or inter-population variability in response to vitamin D supplementation.

VDR affects cholesterol and bile acid synthesis in hepatocytes and serum by controlling expression of genes involved in bile acid synthesis^39^. Results from humans, animals and *in vitro* studies indicated that vitamin D deficiency increases serum cholesterol levels by reducing VDR activity^40^. Disruption of the VDR signaling pathway is associated with a reduction in pancreatic insulin mRNA levels leading to impairment in oral glucose tolerance and reduced insulin secretory capacity in normally fed mice^41^. Several studies showed a correlation between VDR genotypes with risk of obesity, diabetes and metabolic syndrome. However, the information about the effects of vitamin D supplementation and VDR genotypes in improving cardiometabolic aspects in T2DM patients are limited. In the present study, though we found no significant variance in increment 25(OH)D levels among various *Taq-I* and *Bsm-I* genotypic groups after vitamin supplementation, patients with *Taq-I* GG showed significant decrease in LDL-, total cholesterol, triglycerides and HOMA-IR levels than their counterparts. Importantly, except for LDL-cholesterol and HOMA-IR, the association remained significant after Bonferroni adjustments. Similarly, patients carrying *Bsm-I* TT genotypes showed significant differences in LDL-cholesterol and HOMA-IR levels than their counterparts with *Bsm-I* CT and CC genotypes. Hence, despite a similar rise in serum 25(OH)D levels in all subjects after supplementation, patients carrying *Taq-I* GG and *Bsm-I* TT genotypes seem to have better improvement in glycemic and lipid parameters. In particular, *Bsm-I*, *Apa-I*, and *Taq-I* RFLPs are located near the 3′ UTR region, which is known to be involved in the regulation of mRNA stability. Hypothetically, the presence of *Taq-I* G allele and *Bsm-I* T allele might be providing better VDR mRNA stability and half-life resulting to a higher number of VDRs expressed for a longer time in target cells, thus leading in a better response to vitamin D supplementation.

The authors acknowledge several limitations. Findings cannot be generalized since only patients with T2DM were included. Other confounders such as lifestyle, non-genetic factors such BMI, type of anti-diabetic drugs and duration of diabetes were not accounted. Finally, the present study did not have a control/placebo group and therefore bias is likely in the reported changes post intervention.

Nevertheless, VDR polymorphisms influence metabolic response to vitamin D supplementation. VDR genotypic variations might help identify those who will benefit the most from vitamin D treatment. Least improvement in serum 25(OH)D levels was observed in patients carrying VDR *Fok-I* CC genotypes and might need higher doses of vitamin D than their counterparts in order to achieve sufficient levels. Cardiometabolic improvement was more evident in carriers of *Taq-I* GG and *Bsm-I* TT polymorphisms. These carriers may be the better responders to vitamin D therapy in terms of improving metabolic profile and a more tailored approach to vitamin D intervention is warranted. However, further analyses in other ethnic populations are needed to fully validate these findings.

## Material and Methods

### Subjects

This study is a part of our previously described vitamin D intervention study^30^. Briefly, a multi-center, interventional study was setup in the primary health care centers (PHCCs) in Riyadh, Saudi Arabia. 204 T2DM Saudi adults who were mostly vitamin D deficient [serum 25(OH)D < 50 nmol/l)] (men, N = 90: women, N = 114) were given 2000 IU vitamin D3 (Merck Pharma, Germany) daily for 12 months. All subjects answered a generalized questionnaire including present and past medical history. They underwent physical examination and submitted their written informed consents prior to inclusion. Patients taking multivitamins, calcium, cortisone or any other steroids, products with mineral oil, regular antacids, diuretics, phenytoin and phenobarbital medications, weight-loss drugs, gallbladder or gastrointestinal disorders and liver problems, were excluded as well as evidence of metabolic disease (Paget’s disease or osteomalacia), renal stone disease, hyperparathyroidism and abnormal levels of calcium, alkaline phosphatase and phosphorous. Ethical approval of the study including all methods and experimental protocols were obtained from the Institutional Review Board (IRB) of the College of Medicine, King Saud University in Riyadh, Saudi Arabia. All methods were performed in accordance with the relevant guidelines and regulations.

### Anthropometry and blood withdrawal

After an overnight fast, subjects returned to their respective PHCCs for anthropometry and blood withdrawal. Anthropometry included weight, height, hip and waist circumference (cm), systolic and diastolic blood pressure. Body mass index (BMI) was calculated (kg/m^2^). Blood was transferred to a non-heparinized tube for centrifugation. Serum was then aliquoted into plain tubes and stored at −20 °C until further use.

### Biochemical analysis

Methods for biochemical analysis was described in our previous paper^30^. In brief, fasting glucose and lipid profile were measured using chemical analyzer (Konelab, Finland). Serum 25(OH)D levels were measured using ELISA (IDS Ltd., Boldon Colliery, Tyne & Wear, UK).The inter and intra-assay variabilities were 5.3% and 4.6% respectively.. Serum insulin was measured using multiplex assay kits (LuminexW xMAPW Technology platform) (Luminexcorp, Texas). The inter-assay variation was <21% and intra-assay variation was 1.4–7.9%. Vitamin D deficiency was defined as serum 25(OH)D levels < 50 nmol/L. Homeostasis model assessment for insulin resistance (HOMA-IR) was calculated as fasting insulin (IU) x fasting glucose (mmol/L)/22.5.HOMA-β secretion (%) was calculated as 20 × fasting insulin (IU)/(fastingglucose-3.5).

### VDR gene analysis

Genomic DNA was extracted from whole blood using DNeasy blood and tissue kit (Qiagen, Hilden, Germany). The DNA purity (260/280ratio) and concentration were measured using a Nano-drop spectrophotometer. The four VDR SNPs: *Taq-I*(rs731236), *Bsm-I*(rs1544410), *Apa-I*(rs7975232), and *Fok-I*(rs10735810) were evaluated by allelic discrimination real-time PCR using predesigned TaqMan genotyping assays (Applied Biosystems, Foster City, CA). The PCR steps comprisedof a hot start at 95 °C for 10 minutes followed by 45 cycles of 94 °C for 15 seconds and 60 °C for 1 minute; fluorescence detection occurs at of 60 °C. All the genotyping was performed in 10 µl reactions, using TaqMan genotyping mastermix in 96-well plates in an ABI 7000 instrument (Applied Biosystems).

Five samples were excluded from the analysis due to the failure of genotyping results.

### Statistical Analysis

Data was analyzed using SPSS version 21.0, IBM (Armonk, NY, USA). Variables are expressed as mean ± standard deviation (SD). Normality assumption of data was tested using Kolmogrov-Smirnov test. Non-Gaussian variables were logarithmically transformed. Paired T-test was done to compare pre and post supplementation and analysis of variance (ANOVA) to compare genotype groups. Post hoc analysis was done using Tukey’s test. Mantel-Haenzsel test (*χ* ^2^ linear by linear association) for trend for checking p-trend. Bonferroni correction was applied to adjust for multiple comparisons among genotypes. Significance was set at p < 0.05.

## Electronic supplementary material

Supplementary tables
