# Supplementary material for: Vitamin D Receptor Gene Polymorphisms Modify Cardiometabolic Response to Vitamin D Supplementation in T2DM Patients
Source: Sci Rep. 2017 Aug 15;7:8280. doi: 10.1038/s41598-017-08621-7 (PMC5557960; doi:10.1038/s41598-017-08621-7)
Supplement: Supplementary file 1 — Supplementary tables [file 41598_2017_8621_MOESM1_ESM.doc]

**Vitamin D Receptor Gene Polymorphisms Modify Cardiometabolic Response to Vitamin D Supplementation in T2DM Patients**

# Nasser M. Al-Daghri, Abdul Khader Mohammed, Omar S. Al-Attas, Mohammed Ghouse Ahmed Ansari, Kaiser Wani, Syed D. Hussain, Shaun Sabico, Gyanendra Tripathi, Majed S. Alokail

**Supplementary Tables**

**Supplementary Table S1**: Pairwise linkage disequilibrium analysis among *Taq-I, Bsm-I* and *Apa-I* polymorphisms in VDR gene.

| Pairwise LD Correlation | Bsm-I | Apa-I |
| --- | --- | --- |
| Taq-I | 0.87** | -0.47 ** |
| Bsm-I |  | -0.61** |

** indicates p>0.001.

**Supplementary Table S2: Comparisons of the change in variables occurred after 12 months of vitamin d supplementation according to *Taq-I and Bsm-I* haplotypes**

| Parameters | Combination | | | P-values |
| --- | --- | --- | --- | --- |
|  | *Taq-I* GG+*Bsm-I* TT | *Taq-I*AA+ *Bsm-I* CC | *Taq-I*AG+ *Bsm-I* CT |  |
| N | 39 | 48 | 100 |  |
| ΔBMI (Kg/m^2^) | 0.1 ± 0.9 | 0.4 ± 0.9 | 0.1 ± 1.1 | 0.260 |
| Δ Systolic BP (mmHg) | 2.0 ± 15.6 | -2.6 ± 11.0 | 0.7 ± 15.1 | 0.467 |
| Δ Diastolic BP (mmHg) | 1.0 ± 11.3 | -4.3 ± 8.2 | -0.4 ± 11.2 | 0.161 |
| Δ Total Cholesterol (mmol/l) | -0.8 ± 0.9 | -0.3 ± 0.8^A^ | -0.3 ± 0.9^A^ | **0.015** |
| Δ Glucose (mmol/l) # | -1.3 ± 3.6 | 0.4 ± 4.8 | 0.2 ± 4.4^A^ | **0.036** |
| Δ Insulin (uU/ml) # | -2.3 ± 12.0 | 5.5 ± 10.7^A^ | 3.5 ± 11.4^A^ | **0.010** |
| Δ HDL (mmol/l) | -0.1 ± 0.2 | -0.1 ± 0.3 | -0.2 ± 0.3 | 0.141 |
| Δ Triglycerides (mmol/l) | -0.4 ± 0.5 | 0.0 ± 0.7^A^ | -0.1 ± 0.7^A^ | **0.012** |
| Δ HBa1c | -0.7 ± 2.3 | 0.3 ± 2.5 | 0.6 ± 2.4^A^ | **0.030** |
| Δ Vitamin D (nmol/l) | 19.1 ± 16.0 | 23.1 ± 15.7 | 20.1 ± 18.9 | 0.521 |
| Δ LDL (mmol/l) | -0.6 ± 1.0 | -0.2 ± 0.7 | -0.2 ± 0.9 | 0.093 |
| Δ HOMAIR # | -1.6 ± 5.5 | 1.6 ± 5.2 | 1.2 ± 6.9^A^ | **0.024** |

Δ = follow up – Baseline. Data presented as Mean ± SD; # represent non-normal variables. P-values are obtained by using Analysis of Variance (ANOVA) for normal variables and Kruskal-Wallis test for non-normal variables. Superscript A indicates significantly different from *Taq-I* GG+*Bsm-I* TT group.
